# Symptoms of Diseased Elbow-Joint—Removal of Necrosed Bone from the Olecranon—Partial Recovery

**Published:** 1856-09

**Authors:** 


*Symptoms of Diseased Elbow-Joint—Removal of Necrosed Bone from the Olecranon—Partial Recovery.* Under the care of Mr. Curling.

The following are Mr. Langdale's note of the case:—

Emma Simmons was admitted into Sophia Ward, London Hospital, May 31st, 1853. A young woman of short and robust figure, with light hair, blue eyes, and somewhat ruddy complexion, following the occupation of dressmaker. From the county of Norfolk.

The history of the case shows that about ten years ago, while walking in frosty weather, the ground being very icy, she slipped and fell upon the pavement. The greatest part of the injury sustained on this occasion, was borne by the left elbow, and it in a day or two became much swollen and painful in the extreme. Slowly an abscess formed, occupying in its progress two or three months; she then applied to a Surgeon in the neighborhood, but not getting any advantage from the treatment had recourse to, after two or three months more, she relinquished it. Subsequent to the successive formation and maturation of two or three other abscesses about the part, which last all perfectly healed again, it assumed a more or less chronic condition. But the first established abscess left a permanent fistulous aperture, near—and leading down to—the subcutaneous head of the ulna, through which a puriform discharge constantly escaped to the amount of about an ounce per diem. It was not particularly painful during this stage, aching occasionally, and in damp or wet weather, using the patient's own phrase, it was especially "pricking and shooting." For eight years in a somewhat similar state, the arm was in use, in the performance of the duties of a domestic servant, there being no rigidity, and occasionally the discharge ceased for a period. During the past year, however, it again became hot, painful, and swollen. The aching again became intense, and felt as if it arose deep within the bone—was worse at night—prevented rest, and the arm assumed a rigid flexed position. The patient found most alleviation from the use of cold water, constantly applied. The attendant constitutional symptoms, were loss of appetite, feverishness, &c., and excessive debility.

Nevertheless, by resting the arm upon a cushion and leaving the hand free, with the assistance of the sound right arm and hand, (for a week or two at intervals when the symptoms were less severe) she was able to do needlework. It got still worse, and the patient now entered a Hospital, where she remained for three weeks. She states that the limb was proposed for amputation and the patient herself consented; but, through the non-acquiescence of her friends it was not carried into effect, and she left the Hospital in a less favorable position than before.

In about ten days, having procured a ticket of recommendation, she was taken into the London Hospital. After a careful examination of the part, under chloroform, Mr. Curling convinced himself, that in this case, as in the preceding, the disease was limited to the ulna and did not include the joint itself. An operation very similar to that performed in it was accordingly had recourse to, and the trephine having been used, some small fragments of dead bone were removed. The bone was found generally thickened and porous, and it was evident that the disease partook of a strumous character. The patient did remarkably well afterwards. Thenceforward all pain ceased and the thickening gradually subsided. After a while she was sent to the Margate Sea-bathing Infirmary, where she regained her health well.

When last seen, shortly after her return from Margate, there was still an open sinus which led down to bone. The joint, however, was evidently quite free from disease, and the arm in a comparatively useful condition. The strumous nature of the disease of the bone was no doubt the explanation of its not taking on a healing action. There was no reason to suspect that any necrosed fragment remained.—*Med. Times and Gazette.*

